# Mechanical Complications After Acute Myocardial Infarction: A Shock-Stage and Timing-Based Management Framework

**DOI:** 10.3390/jcm15062399

**Published:** 2026-03-21

**Authors:** Caius Glad Streian, Ramona Cristina Novaconi, Iulia Raluca Munteanu, Andrei Raul Manzur, Adrian Grigore Merce, Marciana Ionela Boca, Lucian Silviu Falnita, Ciprian Nicusor Dima, Adrian Petru Merce, Silvius Alexandru Pescariu, Dan Iliescu, Dragos Cozma, Horea Bogdan Feier

**Affiliations:** 1 Department VI Cardiology, Cardiovascular Surgery Clinic, “Victor Babes” University of Medicine and Pharmacy Timisoara, E. Murgu Sq. No. 2, 300041 Timisoara, Romaniahorea.feier@umft.ro (H.B.F.); 2 Institute for Cardiovascular Diseases of Timisoara, Clinic of Cardiovascular Surgery, Gheorghe Adam Street, No. 13A, 300310 Timisoara, Romaniamarciana.boca@umft.ro (M.I.B.);; 3 Doctoral School Medicine-Pharmacy, “Victor Babes” University of Medicine and Pharmacy Timisoara, E. Murgu Sq. No. 2, 300041 Timisoara, Romania; 4 Advanced Research Center, Institute for Cardiovascular Diseases, 300310 Timisoara, Romania; 5 Department of Surgery I—Clinic of Surgical Semiotics & Thoracic Surgery, Center for Hepato-Biliary and Pancreatic Surgery, “Victor Babes” University of Medicine and Pharmacy Timisoara, Eftimie Murgu Square, No. 2, 300041 Timisoara, Romania

**Keywords:** mechanical complications, cardiogenic shock, ventricular septal rupture, free-wall rupture, papillary muscle rupture, mechanical circulatory support

## Abstract

Mechanical complications after acute myocardial infarction (MI)—ventricular septal rupture (VSR), free-wall rupture (FWR), and papillary muscle rupture (PMR)—have become uncommon in the primary percutaneous coronary intervention (PCI) era, yet remain among the most lethal cardiovascular emergencies, with contemporary mortality largely driven by cardiogenic shock and delays to definitive treatment. Although major society documents agree on urgent imaging, early mechanical circulatory support when shock is present, and multidisciplinary decision-making, important transatlantic differences persist, particularly regarding timing of intervention in ventricular septal rupture. This review synthesises current surgical and transcatheter evidence and proposes a unified, physiology-centred framework integrating shock staging, anatomical feasibility, and response to mechanical support. We also introduce STABLE, a structured bedside checklist designed to support consistent daily triage across all three lesions and to align timing decisions with haemodynamic stabilisation rather than centre-specific habit.

## 1. Introduction—The Unresolved Challenge

Mechanical complications following acute myocardial infarction (AMI)—ventricular septal rupture (VSR), left ventricular free-wall rupture (FWR), and papillary muscle rupture (PMR)—have become infrequent in the contemporary reperfusion era, yet they continue to carry a disproportionate mortality burden [1]. Even in modern systems of care, these events often present abruptly with haemodynamic collapse, rapid end-organ hypoperfusion, and a narrow therapeutic window. Outcomes remain poor despite improvements in peri-procedural imaging, intensive care, and the expanding availability of mechanical circulatory support (MCS), underscoring that rarity has not translated into predictability or safety [1,2].

A central challenge is that anatomical disruption alone does not determine prognosis. Registry data consistently show that shock severity at presentation, and its trajectory during early support, drive both operative risk and longer-term survival [2,3,4]. In post-infarction VSR, large surgical datasets demonstrate high operative mortality overall, with substantially better results only when repair can be deferred in selected, stabilised patients—an option unavailable to many because shock progresses before infarct tissue matures [3]. Similarly, outcomes in PMR remain strongly influenced by pre-operative instability, even though surgery is the established definitive therapy [5]. These observations highlight a recurring clinical dilemma: definitive repair is urgent, yet intervention too early can be technically fragile in necrotic myocardium, while waiting can be fatal if shock escalates.

Clinical guidance reflects this tension. Recent European recommendations emphasise early imaging, heart-team governance, and the use of MCS to facilitate stabilisation and individualised timing when feasible [6]. In contrast, North American guidance places stronger emphasis on immediate surgical intervention in VSR regardless of haemodynamic status, while positioning transcatheter approaches mainly as alternatives for prohibitive surgical risk or limited access [7]. Importantly, these differences do not reflect competing biology; rather, they arise from an evidence base dominated by retrospective surgical registries, small interventional series, and limited comparative data [1,3,7].

Recent evidence in acute coronary syndrome populations further underscores the persistent prognostic burden associated with infarct severity, haemodynamic deterioration, and delays in definitive management, reinforcing the need for integrated risk-adapted pathways in post-infarction mechanical complications [8].

The current literature therefore leaves clinicians with two practical needs: first, a coherent framework that integrates shock staging with anatomy and timing across all three lesion types; and second, a structured language that allows heart teams to reassess patients daily, document rationale for delay or escalation, and reduce variability between centres [2,9]. In this structured narrative review, we synthesise contemporary evidence for surgical and transcatheter strategies and propose a unified, physiology-centred algorithm anchored in shock stage and response to MCS [1,2,3]. Within this approach, we introduce the STABLE (Shock stage, Timing from infarction, Anatomical substrate, Biomarkers, Lactate dynamics, Echocardiographic reassessment) Framework as a pragmatic bedside checklist intended to support consistent triage and communication. It is presented as an implementation tool—not as a substitute for multidisciplinary judgement—and is offered to facilitate future harmonised registries and comparative studies [10,11,12].

## 2. Methods

This manuscript was conducted as a structured narrative review of the contemporary literature addressing mechanical complications after acute myocardial infarction, with a focus on ventricular septal rupture, free-wall rupture, and papillary muscle rupture. A literature search was performed in PubMed/MEDLINE and Google Scholar for studies published in English up to June 2025. Search terms included combinations of “mechanical complications myocardial infarction”, “ventricular septal rupture”, “free-wall rupture”, “papillary muscle rupture”, “cardiogenic shock”, “mechanical circulatory support”, “transcatheter closure”, “mitral valve repair”, and “post-infarction rupture”. Priority was given to contemporary guidelines, scientific statements, registry analyses, systematic reviews, meta-analyses, and major observational studies. Reference lists of relevant articles were also screened to identify additional pertinent publications. Case reports and small series were considered selectively when addressing rare procedural strategies or clinically relevant scenarios insufficiently covered by larger datasets. Evidence was synthesised narratively, with emphasis on haemodynamic status, timing of intervention, anatomical feasibility, and multidisciplinary management.

## 3. Pathophysiology and Clinical Phenotypes

Mechanical rupture after AMI represents the extreme end of infarct-related myocardial structural failure. Although contemporary reperfusion has reduced incidence, it has not eliminated the biological cascade that predisposes to tissue disintegration in large, transmural infarctions. Experimental and clinical observations indicate that peak vulnerability typically occurs several days after infarction, when inflammatory infiltration, proteolytic activation, and degradation of extracellular matrix weaken the infarct zone before scar consolidation has occurred [1]. This temporal window explains the classical clustering of rupture events between days 3 and 7, while recognising that very early events still occur in extensive or late-presenting infarctions.

### 3.1. Ventricular Septal Rupture

Post-infarction VSR creates an acute left-to-right shunt, producing abrupt volume overload of both ventricles and a rapid decline in effective systemic cardiac output. The hemodynamic consequence is determined by defect size, location, right ventricular reserve, and systemic vascular resistance. Even moderate defects may precipitate cardiogenic shock if right ventricular function is impaired or systemic vasodilation develops [4]. Surgical registry data demonstrate that mortality correlates more strongly with shock stage than with anatomical characteristics alone [3]. Importantly, tissue friability in the early post-infarct phase contributes to patch dehiscence and residual shunting, which partially explains the improved outcomes observed in selected patients undergoing delayed repair after hemodynamic stabilisation [3,13]. However, delay is only possible when perfusion can be maintained without progressive multiorgan dysfunction.

Transcatheter closure has emerged as a potential alternative in carefully selected cases, particularly when surgical risk is prohibitive or residual defects persist after surgery. Observational series suggest technical feasibility in anatomically suitable defects, but outcomes remain heavily influenced by baseline instability [14,15,16]. Thus, for VSR, anatomy defines feasibility, but shock trajectory determines survival.

### 3.2. Free-Wall Rupture

Left ventricular free-wall rupture represents a distinct biological and clinical entity. Complete “blow-out” rupture typically results in hemopericardium and tamponade with sudden circulatory collapse. In such cases, survival depends on immediate recognition and emergent surgical repair [17,18]. Unlike VSR, there is rarely an opportunity for delayed intervention because hemodynamic deterioration is abrupt and often irreversible without prompt operative control.

Subacute or “oozing” rupture may present with pericardial effusion and evolving tamponade physiology rather than immediate arrest. In these patients, short-term stabilisation with pericardiocentesis and mechanical support may allow controlled transfer and operative planning [19,20]. Nevertheless, even in subacute forms, the window for delay is narrow, and mortality remains high in contemporary series [13]. The biological substrate—extensive transmural necrosis with limited structural support—means that definitive surgical repair is the only durable therapy.

### 3.3. Papillary Muscle Rupture

Papillary muscle rupture leads to acute severe mitral regurgitation with abrupt pulmonary oedema and hemodynamic compromise. The hemodynamic profile differs from VSR in that forward stroke volume may be preserved initially, but effective systemic perfusion declines as regurgitant volume increases. Rapid escalation to shock is common, particularly in complete rupture [5]. Surgical correction remains the standard of care, with contemporary registry data confirming improved survival when prompt intervention is feasible [5,21]. Long-term outcomes are influenced by the choice of repair versus replacement and by pre-operative instability [22,23].

Transcatheter edge-to-edge repair (TEER) has been reported in selected high-risk or inoperable patients. Early registry experience suggests procedural feasibility and hemodynamic improvement, yet mortality remains closely linked to baseline shock stage and organ dysfunction [24,25,26]. As with VSR, interventional success does not neutralise the adverse impact of delayed stabilisation.

Across all three phenotypes, anatomical disruption mandates definitive correction, yet the feasibility and timing of repair are largely determined by hemodynamic trajectory [2,3,4]. This observation underpins a management philosophy in which hemodynamic assessment and support are not adjuncts but central determinants of timing strategy.

**Figure 1:**
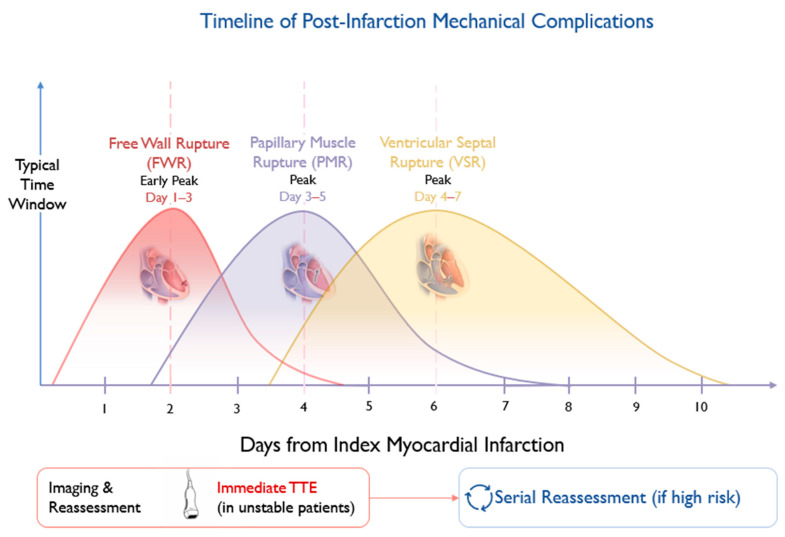
Timeline of post-MI mechanical complications and biological vulnerability window.

The evolving biological substrate following myocardial infarction defines lesion-specific windows of vulnerability and operative risk, as illustrated in Figure 1.

Typical temporal patterns of free-wall rupture (FWR), papillary muscle rupture (PMR), and ventricular septal rupture (VSR) following acute myocardial infarction. The shaded interval reflects the period of maximal myocardial wall fragility. Imaging strategies should be aligned with clinical instability and evolving hemodynamic status.

## 4. Diagnostic Strategy and Structured Reassessment

Early recognition of mechanical complications remains the most decisive modifiable factor in outcome. Although their incidence has declined in the primary PCI era, delayed diagnosis continues to contribute to avoidable deterioration, particularly in patients with large infarctions or evolving hemodynamic instability [1,6]. Diagnosis must therefore be conceptualised not as a single event, but as a dynamic process that integrates clinical suspicion, imaging confirmation, and serial reassessment in parallel with hemodynamic evolution.

### 4.1. Immediate Echocardiographic Assessment

Transthoracic echocardiography (TTE) is the first-line diagnostic modality and should be performed urgently in any post-MI patient presenting with hypotension, new systolic murmur, unexplained pulmonary oedema, or rising lactate [6,7]. Beyond confirming the presence of a defect, echocardiography defines its anatomical characteristics, estimates shunt magnitude or regurgitant severity, and evaluates biventricular function. These parameters directly inform both the feasibility of intervention and the anticipated tolerance of delay.

In VSR, Doppler assessment of shunt flow and estimation of pulmonary-to-systemic flow ratio help quantify hemodynamic impact. In PMR, evaluation of leaflet flail, regurgitant volume, and right ventricular function is essential. In suspected FWR, identification of pericardial effusion with signs of tamponade may be lifesaving. Importantly, hemodynamic interpretation must occur in conjunction with shock staging, as structural findings alone do not predict outcome [2,3].

### 4.2. A Dual-Timepoint Concept

Mechanical rupture is not always evident at initial presentation. Several observational studies demonstrate that defects may evolve over the first days following infarction, particularly in patients with large anterior MI or incomplete reperfusion [1,3]. Accordingly, in selected high-risk patients, a dual-timepoint reassessment strategy may be considered in high-risk patients: immediate imaging at the onset of instability, followed by repeat assessment within 48–72 h if clinical suspicion persists.

This approach reflects the evolving nature of infarct biology during the early post-infarction period. Early negative imaging does not exclude subsequent rupture, especially in patients with persistent chest pain, new hemodynamic changes, or biomarker trajectories suggestive of ongoing myocardial injury [1,6]. Systematic reassessment reduces diagnostic delay and may allow earlier stabilisation before profound shock develops. Although direct comparative evidence for scheduled re-imaging is limited, serial reassessment aligns with contemporary guideline emphasis on early diagnosis and clinical vigilance in unstable patients [6,7].

### 4.3. Advanced Imaging and Procedural Planning

When initial echocardiography confirms a mechanical defect, further imaging may refine procedural strategy. Computed tomography can delineate rupture morphology, tissue thickness, and pericardial anatomy in FWR, supporting surgical planning in selected cases [19,27]. In VSR and PMR, three-dimensional echocardiography and, where feasible, cardiac magnetic resonance imaging (CMR) can clarify defect geometry, rim adequacy, and infarct extension.

However, advanced imaging should not delay definitive intervention in unstable patients. Its role is adjunctive and most valuable when hemodynamic stabilisation has been achieved, allowing time to optimise strategy. As registry analyses consistently show, deterioration in shock stage during diagnostic delay is associated with markedly increased mortality [2,3,4].

### 4.4. Integration with Shock Staging

The SCAI classification provides a structured framework for describing cardiogenic shock severity and progression [2,9]. Integrating imaging findings with SCAI stage at presentation—and during daily reassessment—offers a reproducible method to guide timing decisions. For example, a patient with anatomically suitable VSR but escalating vasopressor requirement and rising lactate represents a fundamentally different scenario from one who is hemodynamically stable with preserved organ perfusion, even if the anatomical defect appears similar.

Thus, diagnostic assessment should not be interpreted in isolation. Structural confirmation, shock stage, biomarker trajectory (including renal and hepatic markers), and response to initial MCS form a composite clinical picture that determines whether immediate intervention, short-term stabilisation, or cautious delay is appropriate [6,7,9].

This diagnostic framework naturally leads to the core question of management: how should timing and mechanical support be aligned with lesion type and shock trajectory?

**Figure 2:**
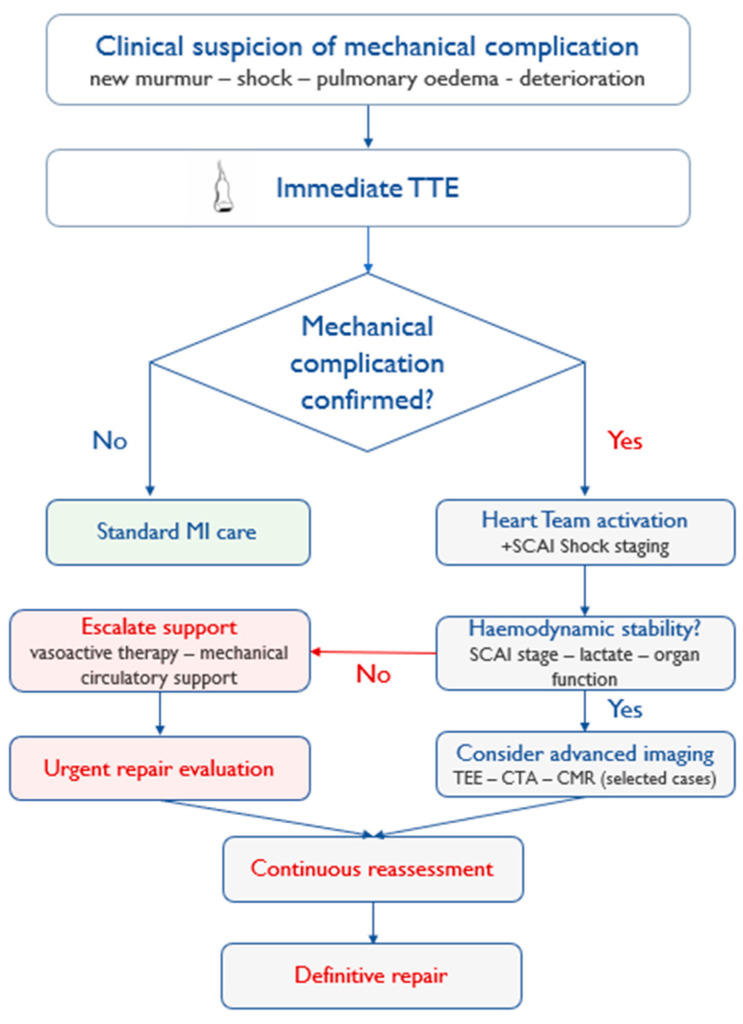
Pragmatic imaging and reassessment pathway in suspected post-MI mechanical complications.

A pragmatic, escalation-based imaging strategy integrating clinical instability and hemodynamic evolution is summarised in Figure 2. A concise overview of phenotype-specific presentation and diagnostic hallmarks is provided in Appendix A.

Stepwise imaging strategy beginning with immediate TTE in unstable patients, followed by targeted escalation to TEE, CTA, or CMR as clinically indicated. Serial reassessment is recommended in high-risk or hemodynamically evolving cases.

## 5. A Shock-Guided Therapeutic Framework

We propose a structured, shock-guided therapeutic framework integrating anatomy, physiology, and timing in post-MI mechanical complications (Graphical Abstract).

Management of post-infarction mechanical complications requires a sequential logic that reconciles two competing risks: intervention in structurally fragile myocardium versus deterioration under ongoing shock. Across contemporary registries, hemodynamic instability consistently outweighs anatomical variables as the dominant predictor of mortality [2,3,4]. Accordingly, therapeutic strategy should be structured around three consecutive questions: Is shock present? Can perfusion be stabilised? Is there a safe window for definitive repair?

### 5.1. Ventricular Septal Rupture

In VSR, cardiogenic shock results from acute left-to-right shunting, reduced forward output, and progressive ventricular failure. Surgical repair remains the standard definitive therapy, yet operative mortality in large series exceeds 40% when performed early in unstable patients [3,28]. Observational analyses and meta-analytic data suggest lower operative mortality when surgery is deferred beyond the first week in selected patients who achieve hemodynamic stabilisation [3,27]. However, this apparent benefit must be interpreted cautiously because delayed-repair cohorts are inherently affected by survivorship and selection bias: only patients who remain hemodynamically stable enough to survive the early post-infarction period can ultimately undergo delayed repair. Therefore, improved outcomes in delayed cohorts should not be interpreted as evidence that postponement is universally safer, but rather that delay may be advantageous only in carefully selected patients with preserved or restored organ perfusion.

In patients presenting in advanced shock (SCAI Class D or E), immediate stabilisation with mechanical circulatory support is often required [2,9]. Intra-aortic balloon pump may reduce afterload and shunt fraction modestly, but more profound support, including veno-arterial extracorporeal membrane oxygenation or combined unloading strategies, may be necessary when shock persists [10,29]. Failure to achieve hemodynamic improvement within hours rather than days should prompt definitive intervention, as progression of shock markedly worsens operative risk [3,4].

Transcatheter closure has emerged as an alternative in anatomically suitable defects or in patients deemed prohibitive surgical risk. Meta-analyses and registry data demonstrate technical feasibility, but survival remains closely tied to baseline shock severity rather than procedural modality [14,15,16]. Thus, device therapy should be viewed as a complementary strategy rather than a substitute for timely hemodynamic stabilisation.

Compared with surgery, percutaneous VSR closure may offer lower immediate procedural invasiveness, avoidance of sternotomy and cardiopulmonary bypass, and a potential bridging or salvage option in selected patients with prohibitive surgical risk, residual post-surgical shunts, or anatomically suitable defects. However, its limitations remain substantial: device anchoring may be challenging in friable necrotic septal tissue, complex or serpiginous defects may be unsuitable, residual shunting is frequent, and procedural success remains strongly dependent on defect morphology and baseline hemodynamic status. By contrast, surgery offers direct visual assessment, debridement of necrotic tissue, and the possibility of more definitive structural repair, but at the cost of high perioperative mortality when undertaken in unstable patients early after infarction. Therefore, the choice between surgical and transcatheter treatment should be individualised by the heart team according to shock stage, defect anatomy, tissue quality, institutional expertise, and the possibility of achieving adequate hemodynamic stabilisation before repair.

Taken together, these data support a physiology-driven approach in which timing of VSR repair is guided not solely by days from infarction, but by the trajectory of shock and the capacity to maintain organ perfusion under support [2,3].

In patients considered for transcatheter treatment of VSR, coronary anatomy should be reviewed systematically, and revascularization strategy should be incorporated into the definitive treatment plan. Whenever anatomically feasible and clinically appropriate, complete or functionally meaningful revascularization—by PCI or CABG—should accompany or follow structural repair as part of an integrated heart-team strategy rather than be considered separately from defect closure.

A comparative overview of early versus delayed repair in post-infarction VSR is provided in Table 1, with emphasis on absolute mortality patterns, shock-related selection, and the interpretive limitations of retrospective datasets.

### 5.2. Free-Wall Rupture

In contrast to VSR, free-wall rupture generally offers limited opportunity for delay. Complete rupture with tamponade requires emergent surgical repair, as mortality is extremely high without emergent intervention [17,30]. Mechanical support may facilitate transfer or brief stabilisation, but it cannot substitute for definitive repair.

Subacute forms may allow controlled hemodynamic stabilisation, particularly when rupture is contained and tamponade evolves more gradually [19,27]. Even in these cases, however, postponement is measured in hours to a few days rather than weeks. Surgical series emphasise that early recognition and rapid operative management are the principal determinants of survival [13,27]. Consequently, the timing dilemma central to VSR is less applicable to FWR, where biological instability predominates.

### 5.3. Papillary Muscle Rupture

Papillary muscle rupture produces acute severe mitral regurgitation and rapid pulmonary congestion. Although systemic output may initially appear preserved, progressive hemodynamic collapse frequently ensues. Contemporary registry data confirm that early surgical intervention remains the cornerstone of therapy and is associated with improved survival when performed before irreversible multiorgan dysfunction develops [5,21].

Unlike VSR, delay for tissue maturation is rarely appropriate in complete PMR given the potential for rapid hemodynamic collapse [5,21].

Mechanical circulatory support can stabilise patients with severe regurgitation and shock, particularly when pulmonary oedema and hypoxaemia complicate the clinical course. However, as in VSR, prolonged delay without hemodynamic improvement increases operative mortality [5]. Observational experience with transcatheter edge-to-edge repair (TEER) suggests feasibility in selected high-risk or inoperable patients, but outcomes remain strongly influenced by baseline shock stage and end-organ function [24,25,26].

In PMR patients undergoing transcatheter treatment, coronary anatomy and the feasibility of complete revascularization should be explicitly assessed within the heart-team discussion. Structural correction of acute mitral regurgitation does not eliminate the need to address the ischaemic substrate, and PCI or CABG should be planned whenever appropriate to optimise myocardial recovery and overall clinical outcome.

Across VSR, FWR, and PMR, a consistent principle emerges: anatomical repair is definitive, yet survival is governed by hemodynamic trajectory. Mechanical support should therefore be used strategically—to create a window for safer intervention when possible, and to avoid futile delay when stabilisation cannot be achieved [2,10].

## 6. Mechanical Circulatory Support: Principles of Escalation and Monitoring

Mechanical circulatory support (MCS) occupies a central position in the management of post-infarction mechanical complications complicated by cardiogenic shock. Its purpose is not curative; rather, it serves to stabilise systemic perfusion, limit ongoing organ injury, and—when feasible—create a safer window for definitive repair. Registry analyses indicate that hemodynamic status at the time of intervention strongly influences operative risk and survival [2,3,4]. Consequently, the decision to initiate and escalate support should be guided by objective hemodynamic targets and dynamic reassessment rather than by lesion type alone.

### 6.1. Initial Support Strategies

Intra-aortic balloon pump (IABP) has historically been used in VSR and PMR to reduce afterload and improve coronary perfusion. Although randomised data in unselected cardiogenic shock populations failed to demonstrate mortality benefit [29], its physiological effects—afterload reduction and modest augmentation of forward flow—may be beneficial in selected mechanical complications, particularly when shunt fraction or regurgitant volume is sensitive to changes in ventricular loading conditions. Nevertheless, IABP alone is frequently insufficient in advanced shock.

When hypotension persists with escalating vasopressor requirements, veno-arterial extracorporeal membrane oxygenation (VA-ECMO) provides full cardiopulmonary support and rapid restoration of systemic perfusion. Data from registry cohorts of patients with post-infarction mechanical complications suggest that VA-ECMO can serve as an effective bridge to surgery in selected unstable patients [10]. However, VA-ECMO increases left ventricular afterload and may exacerbate pulmonary congestion or shunt flow unless combined with unloading strategies. Therefore, its use should be accompanied by careful monitoring of ventricular distension and pulmonary pressures.

Percutaneous ventricular assist devices that directly unload the left ventricle may reduce left ventricular pressures and potentially decrease shunt flow in selected patients, although current evidence remains observational [2]. In PMR with severe mitral regurgitation, unloading may alleviate pulmonary oedema but does not eliminate the need for definitive correction.

### 6.2. Defining Response and Escalation

The clinical value of MCS depends on whether it achieves meaningful hemodynamic recovery. Objective parameters should therefore guide daily assessment. Restoration of cardiac index to ≥2.2–2.4 L/min/m^2^, reduction in serum lactate toward normal values, decreasing vasopressor requirement, and stabilisation of renal function collectively indicate effective perfusion [2,4]. Persistent hyperlactataemia or escalating catecholamine support despite adequate device flow signals inadequate circulatory support or irreversible shock progression.

Importantly, failure to demonstrate hemodynamic improvement within a short and predefined timeframe—often hours rather than days in advanced shock—should prompt reconsideration of strategy. Prolonged delay under ineffective support is associated with worsening operative risk and diminished survival [3,4]. Thus, escalation decisions should be proactive rather than reactive.

### 6.3. MCS as Bridge, Not Destination

In the context of mechanical rupture, MCS is rarely definitive therapy. Its role is to bridge to surgical or transcatheter correction, or in selected cases, to palliation, when repair is not feasible. Data from extracorporeal support registries indicate that outcomes are most favourable when MCS is implemented early in the shock trajectory rather than as rescue after cardiac arrest or refractory multiorgan failure [10]. Integration of shock staging using the SCAI framework allows structured documentation of progression and may support more timely escalation [2,10].

Taken together, these principles reinforce a central concept: mechanical support must be embedded within a time-sensitive decision pathway. The goal is not simply to maintain circulation, but to determine whether stabilisation sufficient for safer repair can be achieved. If so, delay may improve technical durability in selected VSR cases; if not, definitive intervention should not be postponed in the face of escalating shock [3,28].

## 7. The STABLE Framework: A Structured Framework for Bedside Triage

Clinical decision-making in post-infarction mechanical complications is often influenced by centre experience and individual judgement, particularly when evidence is limited and recommendations diverge [6,7]. While multidisciplinary discussion is universally endorsed, there remains variability in how shock severity, anatomical features, biomarker trajectory, and timing from infarction are weighed in daily practice. To facilitate structured reassessment and improve consistency in communication, we propose the STABLE Framework as a practical bedside checklist integrating these domains.

The STABLE Framework incorporates six components: Shock stage, Timing from infarction, Anatomical suitability—including defect morphology, ventricular involvement, and coronary anatomy/revascularization status—Biomarker trend, Lactate dynamics, and Echocardiographic or imaging morphology. Each element reflects variables repeatedly associated with operative risk and survival in registry analyses and contemporary scientific statements [1,2,3,9]. Importantly, the STABLE Framework is not a validated predictive or prognostic score and does not generate a numeric risk estimate. It is not intended to replace heart-team deliberation, but rather to provide a reproducible structure for documenting physiological evolution and guiding timing discussions.

We propose the STABLE framework as a structured bedside reassessment tool integrating shock stage, timing, anatomy, biomarkers, lactate dynamics, and echocardiographic evolution. The components of the STABLE framework are summarised in Table 2.

### 7.1. Shock Stage

Shock severity, classified according to the SCAI framework, consistently correlates with mortality across mechanical complication cohorts [2,3]. Progression from early hypotension to advanced shock with end-organ dysfunction markedly increases operative risk. Incorporating shock stage as the first domain of STABLE ensures that anatomical decisions are interpreted within a physiological context rather than in isolation [9].

### 7.2. Timing from Infarction

Time since infarction influences myocardial integrity and surgical durability. Observational data in VSR suggest improved outcomes when repair is deferred beyond the acute inflammatory phase in stabilised patients [3,27]. However, this association is strongly influenced by survivorship and selection bias, as delayed repair is feasible only in patients who remain hemodynamically stable long enough to reach intervention. Conversely, in FWR and complete PMR, delay may be unsafe due to abrupt hemodynamic collapse [5,12]. Including timing as an explicit variable acknowledges these lesion-specific differences while reinforcing that delay is contingent on hemodynamic stability rather than time alone.

### 7.3. Anatomical Suitability

Defect size, rim quality, ventricular function, associated valvular or right ventricular involvement, and the underlying coronary anatomy influence both feasibility and procedural choice. In practice, anatomical suitability should not be limited to the mechanical lesion itself, but should also include the revascularization strategy, particularly in patients considered for transcatheter treatment. Registry and meta-analytic data demonstrate that anatomical complexity impacts residual shunting and mortality after VSR repair or closure [13,14]. Structured documentation of anatomical suitability therefore supports transparent decision-making between surgical and transcatheter strategies while ensuring that defect repair and myocardial revascularization are planned as part of the same definitive pathway.

### 7.4. Biomarker Trajectory and Lactate Dynamics

Rising serum lactate and worsening renal or hepatic function reflect inadequate perfusion and predict adverse outcomes in cardiogenic shock [2,4]. Persistent hyperlactataemia despite MCS suggests ongoing tissue hypoxia and may indicate the need for urgent definitive intervention rather than continued delay. Including lactate and biomarker trends within STABLE emphasises that physiological response over time is as important as initial presentation.

### 7.5. Imaging Morphology

Serial imaging allows assessment of defect evolution, ventricular function, and response to unloading. In VSR, repeat evaluation may clarify rim maturation; in FWR, progression of pericardial effusion may signal impending tamponade; in PMR, worsening regurgitation can herald hemodynamic collapse [5,19]. Incorporating imaging reassessment formalises the principle of dynamic evaluation.

Collectively, the STABLE Framework provides a shared language for heart-team deliberation. Rather than dictating intervention thresholds, it encourages daily documentation of whether stabilisation targets have been achieved and whether conditions favour delay or mandate escalation. In this sense, the STABLE Framework functions as an implementation tool aligned with contemporary calls for structured shock staging and quality indicators in acute cardiac care [9,10,11].

Prospective validation will be necessary before formal prognostic inference can be drawn. Nonetheless, by operationalising variables already recognised in guidelines and registries, the STABLE framework may reduce unwarranted variability and support future harmonised registries [1,6,10].

The STABLE framework is intended to be completed at baseline and repeated at least daily, or after major clinical changes. Worsening shock stage, rising lactate, or deteriorating biomarker trajectory despite mechanical support should prompt reconsideration of delay and favour timely definitive intervention. Conversely, demonstrable hemodynamic improvement and stabilisation may support short postponement in selected VSR cases when anatomical conditions are favourable [2,3,9].

Selected high-risk markers and escalation considerations that may inform multidisciplinary decision-making are summarised in Appendix B [2,3,31,32,33,34,35,36].

## 8. Harmonisation, Systems of Care, and Future Directions

The management of post-infarction mechanical complications illustrates a broader challenge in acute cardiovascular medicine: rare but catastrophic conditions for which high-quality randomised evidence is scarce and clinical practice is shaped largely by observational registries and institutional experience [1,6]. Divergences between contemporary European and North American recommendations, particularly regarding the timing of intervention in VSR, reflect these evidentiary limitations rather than fundamentally different biological principles [6,7].

The present review seeks to advance beyond prior lesion-specific or predominantly anatomy-driven approaches by proposing a unified physiology-based framework applicable across VSR, FWR, and PMR. Rather than focusing exclusively on structural diagnosis or guideline-mandated urgency, this framework integrates shock severity, biological timing, response to mechanical circulatory support, and repeated multidisciplinary reassessment into a single decision pathway. In this respect, the review does not replace existing recommendations but extends them by offering a practical structure for bedside triage in situations where anatomy alone does not adequately define timing or operative readiness.

In this context, harmonisation does not necessarily require uniform procedural strategy, but rather alignment in how patients are assessed, staged, and re-evaluated. Standardised shock classification using the SCAI framework has already improved the clarity of communication in cardiogenic shock and provides a reproducible language for clinical documentation and research [2,9]. Embedding such structured staging into daily practice, alongside predefined hemodynamic targets and serial imaging reassessment, may reduce unwarranted variability in timing decisions.

Quality indicators developed for acute coronary syndromes emphasise early diagnosis, multidisciplinary governance, and appropriate escalation of care [12]. Applying these principles to mechanical complications supports the development of regionalised systems in which complex cases are rapidly transferred to centres with surgical and advanced mechanical support capability. Observational data from specialised networks suggest that outcomes improve when expertise and volume are concentrated [11]. In this setting, hub-and-spoke models with predefined transfer pathways may be particularly relevant for rare but high-risk conditions such as VSR, FWR, and PMR. Recent acute coronary syndrome data also highlight that prognosis remains strongly influenced by clinical presentation, hemodynamic compromise, and downstream treatment strategy, supporting the need for structured systems of care and timely escalation pathways in patients at risk for post-infarction mechanical complications [8].

Future progress will depend less on isolated case series and more on collaborative, registry-embedded research. Large contemporary datasets, including extracorporeal support registries, have already demonstrated the value of multicentre aggregation in refining patient selection and timing strategies [10]. Extending such collaborative frameworks to include standardised documentation of shock stage, timing intervals, anatomical characteristics, and support strategies would permit more robust comparative analyses. Structured implementation tools, such as the STABLE framework, may facilitate such harmonisation by ensuring that key physiological variables are consistently captured across centres.

Ultimately, reducing mortality in post-infarction mechanical complications will require integration rather than innovation alone: early recognition, disciplined shock staging, appropriate mechanical support, timely definitive repair, and system-level coordination. While definitive randomised trials may remain challenging in this rare population, harmonised registries and adaptive observational studies offer a realistic path forward [1,6,10].

## 9. Conclusions

Mechanical complications after acute myocardial infarction remain among the most lethal emergencies in contemporary cardiology. Although their incidence has declined, mortality continues to be driven primarily by cardiogenic shock and delays in definitive management [1,2,3]. Across ventricular septal rupture, free-wall rupture, and papillary muscle rupture, anatomical correction is essential, yet survival is determined largely by hemodynamic trajectory and the capacity to stabilise organ perfusion.

A management strategy anchored in structured shock staging, objective assessment of response to mechanical circulatory support, and careful alignment of timing with infarct biology offers a pragmatic and reproducible framework [2,9]. By integrating these elements across all three lesion types, clinicians may reduce therapeutic ambiguity and improve consistency of care.

The STABLE Framework is proposed as a bedside implementation tool to support daily reassessment and heart-team communication rather than as a predictive model. It should be interpreted as a structured clinical framework and not as a validated risk stratification score. Prospective validation and multinational registry collaboration will be necessary to refine thresholds and optimise patient selection [10,11,12]. Until such data emerge, disciplined physiology-guided triage remains the most defensible approach in this high-risk population.

## Figures and Tables

**Table 1:** Early versus delayed repair in post-infarction ventricular septal rupture: mortality patterns, shock-related considerations, and interpretive limitations.

Study/Dataset	Early Repair	Delayed Repair	Shock-Related Interpretation/Key Limitation
[3]	Higher operative mortality when repair was performed within 7 days after MI; mortality in the earliest surgical window was approximately 55%	Lower operative mortality when surgery was delayed beyond 7 days; approximately 20%	The apparent benefit of delayed repair is strongly confounded by survivorship and selection bias, because only patients who remain hemodynamically stable long enough can undergo later surgery
[28]	Early surgery was associated with significantly higher mortality; pooled early mortality remained approximately 50% or higher in most series	Delayed surgery was associated with lower pooled mortality, often approximately 20–30%	Meta-analytic findings are limited by retrospective data, heterogeneous definitions of “early” and “delayed,” and underreporting of shock severity
[9]	Outcomes were particularly poor in patients presenting with cardiogenic shock, especially with persistent hypoperfusion and multiorgan dysfunction	Delay was feasible only in the subset achieving hemodynamic stabilisation under support	Shock stage is a major confounder: timing cannot be interpreted independently from hemodynamic trajectory
[3,13]	Early intervention is often unavoidable in unstable patients, despite high mortality, because progressive shock precludes waiting for tissue maturation	Delayed repair may improve technical durability in selected stabilised patients	Delayed cohorts are not directly comparable with early cohorts because they represent a pre-selected survivor population

Interpretive note: Reported mortality estimates vary across datasets because definitions of early versus delayed repair are not fully uniform, and comparisons are substantially influenced by shock stage and survivorship bias.

**Table 2:** STABLE framework for structured bedside reassessment in post-infarction mechanical complications, integrating shock stage, timing, anatomical suitability, biomarker trends, lactate dynamics, and serial imaging. The framework is intended to support clinical reassessment and multidisciplinary discussion and is not validated for prognostic prediction.

Domain	Clinical Focus
Shock stage	SCAI classification
Timing	Time from index myocardial infarction
Anatomy	Defect morphology, ventricular involvement, coronary anatomy, and revascularization feasibility
Biomarkers	Organ dysfunction markers (renal, hepatic)
Lactate	Perfusion adequacy and dynamic trend
Echo	Serial reassessment of lesion, ventricular function and hemodynamics

## Data Availability

This article is a narrative review based on previously published studies. No new data were generated or analysed.

## Abbreviations

The following abbreviations are used in this manuscript:

AMI	acute myocardial infarction
CMR	cardiovascular magnetic resonance
CTA	computed tomography angiography
ECMO	extracorporeal membrane oxygenation
FWR	free-wall rupture
IABP	intra-aortic balloon pump
MCS	mechanical circulatory support
MI	myocardial infarction
PCI	percutaneous coronary intervention
PMR	papillary muscle rupture
SCAI	Society for Cardiovascular Angiography and Interventions
STABLE	Shock stage, Timing from infarction, Anatomical suitability, Biomarker trend, Lactate dynamics, Echocardiographic reassessment
TEE	transoesophageal echocardiography
TEER	transcatheter edge-to-edge repair
TTE	transthoracic echocardiography
VA-ECMO	veno-arterial extracorporeal membrane oxygenation
VSR	ventricular septal rupture

## Appendix A

**Table A1:** Recognition and clinical presentation of post-MI mechanical complications.

Phenotype	Typical Onset	Hallmark Imaging	Red-Flag Clinical Sign
VSR	Days 3–5 (PCI era); 7–10 (pre-PCI)	High-velocity left-to-right jet on TTE or TEE; septal discontinuity on CMR	New harsh holosystolic murmur ± thrill
FWR	Days 1–3 (blow-out); 4–7 (subacute)	Pericardial effusion or wall defect on TTE/TEE or CTA; contrast extravasation on CTA	Sudden hypotension, pulsus paradoxus, electromechanical dissociation
PMR	Days 2–7 (infero-posterior MI)	Flail posterior leaflet with severe mitral regurgitation on TEE or CMR	Flash pulmonary oedema and new systolic murmur

## Appendix B

**Table A2:** Risk markers and escalation considerations in post-MI mechanical complications. Summary of selected clinical and hemodynamic markers associated with adverse outcomes in post-infarction mechanical complications. Thresholds are intended to support clinical judgement and multidisciplinary discussion and do not represent prescriptive treatment recommendations.

Metric/Score	High-Risk Threshold	Prognostic Impact (30-Day or Operative Mortality)	Suggested Clinical Consideration	Primary Reference
**Killip–Kimball**	Class ≥ III	Associated with increased early mortality	Consider reassessment of shock stage and repeat imaging	[30]
**SCAI shock**	C → D/E progression	Associated with markedly increased in-hospital mortality	Consider early mechanical circulatory support and advanced imaging	[2]
**EuroSCORE II**	>20	Associated with increased operative risk in mechanical rupture cohorts	Consider referral to tertiary centre and multidisciplinary timing strategy	[31]
**STS surgical-VSR models**	Incorporating defect size and lactate	Associated with improved risk discrimination in surgical VSR cohorts	May assist multidisciplinary timing decisions in selected patients	[3]
**INTERMACS profile**	1–2 (crash/progressive)	Associated with poor short-term survival without mechanical support	Consider early MCS evaluation	[32]
**IABP-SHOCK II criteria**	MAP < 65 mm Hg despite vasopressors	Hemodynamic improvement without survival benefit	May be considered when advanced MCS is unavailable	[28,34]
**RV/LV diameter ratio on echocardiography**	≥1.0	Associated with increased early mortality	Consider urgent multidisciplinary reassessment	[35]
